# Criteria for the authorisation of specialty training for competence in internal medicine and rheumatology—a position paper of the German Society for Rheumatology and Clinical Immunology

**DOI:** 10.1007/s00393-025-01618-y

**Published:** 2025-02-27

**Authors:** Alexander Pfeil, Martin Fleck, Martin Aringer, Xenofon Baraliakos, Diana Ernst, Isabell Haase, Christiana Hillebrecht, Bimba Franziska Hoyer, Gernot Keyßer, Ina Kötter, Andreas Krause, Martin Krusche, Hanns-Martin Lorenz, Fabian Proft, Florian Schuch, Diana Vossen, Anna Voormann, Ulf Wagner, Jürgen Wollenhaupt, Christof Specker

**Affiliations:** 1 Department of Internal Medicine III, Rheumatology Centre (according to GBA criteria) and Section of Rheumatology and Osteology, Jena University Hospital, Friedrich Schiller University Jena, Am Klinikum 1, 07747 Jena, Germany; 2 Department of Internal Medicine I, University Hospital Regensburg, Regensburg, Germany; 3 Clinic and Polyclinic for Rheumatology/Clinical Immunology, Asklepios Klinikum Bad Abbach, Bad Abbach, Germany; 4 Department of Rheumatology, Medical Clinic and Polyclinic III, University Hospital Carl Gustav Carus of the Technical University of Dresden, Dresden, Germany; 5 Ruhr University Bochum, Rheumazentrum Ruhrgebiet Herne, Germany; 6 Division of Rheumatology and Systemic Inflammatory Diseases, III Department of Medicine, University Medical Center Hamburg-Eppendorf, Hamburg, Germany; 7 Clinic for Internal Rheumatology, Red Cross Hospital, Bremen, Germany; 8 Section Rheumatology, Clinic for Internal Medicine I, University Hospital Schleswig-Holstein, Kiel, Germany; 9 Department of Internal Medicine, Clinic for Internal Medicine II, University Hospital Halle, Halle (Saale), Germany; 10 Department of Rheumatology, Clinical Immunology and Osteology, Immanuel Krankenhaus Berlin, Berlin, Germany; 11 Division of Rheumatology, Medical Clinic V, University Hospital Heidelberg, Heidelberg, Germany; 12 Department of Gastroenterology, Infectiology and Rheumatology (including Nutrition Medicine), Charité—Universitätsmedizin Berlin, corporate member of Freie Universität Berlin and Humboldt-Universität zu Berlin, Berlin, Germany; 13 Internal medicine practice, rheumatology/nephrology, Erlangen, Germany; 14 German Society for Rheumatology and Clinical Immunology, Berlin, Germany; 15 Department of Rheumatology, Clinic and Polyclinic for Endocrinology, Nephrology, Rheumatology, University Hospital Leipzig, Leipzig, Germany; 16 Immunologikum Hamburg, Hamburg, Germany; 17 Department of Rheumatology and Clinical Immunology, Evangelisches Krankenhaus Kliniken Essen-Mitte, Essen, Germany; 18 Rheumatology & Immunology, Hannover Medical School, Hannover, Germany

**Keywords:** Model training regulations, Training content, Training plan, Medical specialist training, Medical specialist qualification, Musterweiterbildungsordnung, Weiterbildungsinhalte, Weiterbildungsplan, Facharztweiterbildung, Facharztbezeichnung

## Abstract

The model advanced training regulations define the content of advanced training to achieve the qualification of medical specialist in all specialties and sub-specialties of medicine. As rheumatology is one of the ten specialties for internal medicine in Germany, regulations cover basic competencies of general and all other specialties in internal medicine as well as special skills in rheumatology. There are currently no criteria for issuing the authorization in advanced training. This position paper describes the criteria proposed by the German Society for Rheumatology and Clinical Immunology (DGRh), which should be the foundation for the issuance of authorization for advanced training in the field of internal medicine and rheumatology and for the assessment of the duration. The model advanced training regulations 2018 and the advanced training plan recommended by experts function as the basis for this. Based on the criteria, the authorization for advanced training to advanced specialist training in internal medicine and rheumatology can be allocated in a standardized, graded and transparent manner throughout Germany. This enables an optimal quality of advanced training in rheumatology, which can be adapted to the future developments in the discipline.

## Abstract (de)

Die Musterweiterbildungsordnung definiert die Weiterbildungsinhalte zur Erlangung der Facharztbezeichnung in allen Fachgebieten und Teilgebieten der Medizin. Die Rheumatologie ist eines der zehn Fachgebiete für Innere Medizin in Deutschland, werden in der Musterweiterbildungsordnung die Grundkompetenzen aller internistischen Fachgebiete sowie die besonderen Kompetenzen der Rheumatologie geregelt. Kriterien zur Erteilung der Weiterbildungsbefugnis liegen nicht vor. Das Positionspapier beschreibt die von der Deutschen Gesellschaft für Rheumatologie und Klinische Immunologie (DGRh) vorgeschlagenen Kriterien, die bei der Erteilung einer Weiterbildungsbefugnis im Gebiet Innere Medizin und Rheumatologie und für die Bemessung ihres zeitlichen Umfangs zugrunde gelegt werden sollten. Dabei fungieren die Musterweiterbildungsordnung 2018 und der fachlich empfohlene Weiterbildungsplan als Basis. Anhand der Kriterien kann die Weiterbildungsbefugnis zur Facharztweiterbildung Innere Medizin und Rheumatologie standardisiert, abgestuft und transparent im gesamten Bundesgebiet vergeben werden. So wird eine qualitätsoptimierte Weiterbildung in der Rheumatologie ermöglicht, die an zukünftige Entwicklungen des Faches angepasst werden kann.

The field of rheumatology focuses on systemic diseases which are characterised by immune-mediated acute or chronic recurrent inflammation in the musculoskeletal and various other organ systems [2]. In addition, rheumatologists also treat non-inflammatory rheumatic and musculoskeletal diseases [13], although in Germany, the emphasis is on inflammatory rheumatic diseases due to the capacities available in rheumatology, and the position paper is based on the situation in Germany.

Considering the demographic development in Germany, an increase in the incidence and prevalence of inflammatory rheumatic diseases is to be expected in the next few years [1]. Due to the already existing shortage of specialists in the out- and inpatient sectors [2], as well as the impending generational change in rheumatology [6], successful specialty training to raise specialists in internal medicine and rheumatology is essential for optimal patient care.

The content of the training for a specialist in internal medicine and rheumatology is based on the model specialty training regulations (*Musterweiterbildungsordnung*, MWBO) and the specification of the training content in the recommended specialty training plan (*Fachlich empfohlener Weiterbildungsplan,* FEWP) [4, 5]. According to the 2018 model training regulations, at least 72 months must be spent in internal medicine, with 36 months of specialty training planned for the subspecialty of internal medicine and rheumatology. Based on the MWBO, at least 24 months of this time must be spent in inpatient care [4]. However, some German State Chambers of Physicians (*Landesärztekammern*) have deviated from this regulation when implementing it. This was justified by the lack of inpatient training positions, which could lead to a bottleneck in training opportunities for rheumatologists [2].

The MWBO and the FEWP define in detail the competences and training content for the qualification in internal medicine and rheumatology at the level of the trainee. By contrast, the qualification of the training supervisors is not specified, so that to date, there are no recommendations for the granting and scope (12 months, 24 months or 36 months) of the authorisation to provide training in the field of internal medicine and rheumatology (see Fig. 1).

**Fig. 1:**
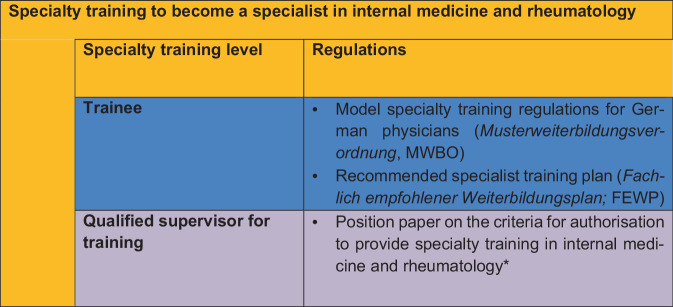
Levels of specialty training to become a specialist in internal medicine and rheumatology, with an outline of the regulations (asterisk: future classification of the position paper regarding the criteria for authorisation to provide specialty training for specialists in internal medicine and rheumatology)

This position paper therefore proposes criteria for granting authorisation to provide specialty training in internal medicine and rheumatology, which should make the process of granting authorisation transparent and standardised and ensure the quality of specialty training.

## Criteria for granting authorisation for specialty training for specialist competence in internal medicine and rheumatology

The following eligibility criteria have been developed for granting authorisation for specialty training. The criteria are based on the training blocks mentioned in the MWBO and the FEWP [4, 5]. The criteria for granting authorisation for specialty training represent the minimum requirements for those authorised to provide training.

### I. Timeframe

The time basis for granting authorisation for specialty training in the field of internal medicine and rheumatology is built on the MWBO [4]. To be awarded a specialist qualification in internal medicine and rheumatology, a period of 72 months must be completed in the field of internal medicine, of which 36 months must be in the subspecialty of internal medicine and rheumatology [4]. Furthermore, the criteria for granting authorisation for specialty training in internal medicine and rheumatology relate to the 36-month period in the subspecialty of internal medicine and rheumatology.

### II. Specification of the specialty training content (see Fig. 2)

The content of the training is specified based on the MWBO 2018 and the FEWP. Each specific training topic is assigned a competence number, which must be taught by the training supervisors. Of the 33 competences developed in this manner, the training topics with the competence numbers 3, 8 and 30 are classified as mandatory for a full rheumatology training authorisation of 36 months. The differentiation into a 12-month, 24-month or full training authorisation is based on the existing skills at the training centre.

**Fig. 2:**
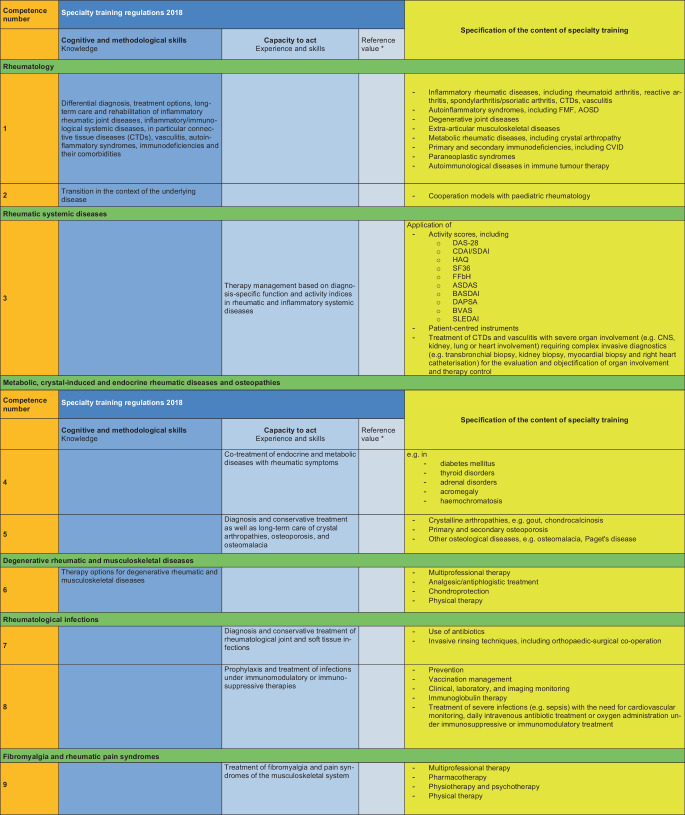
Specification of the content of specialty training with reference to the specific content of specialty training in internal medicine and rheumatology. Asterisk: this position paper deliberately avoids mentioning reference numbers, which may be used, if necessary, to determine the number of specialty training positions at a specialty training centre

**Fig. 2:**
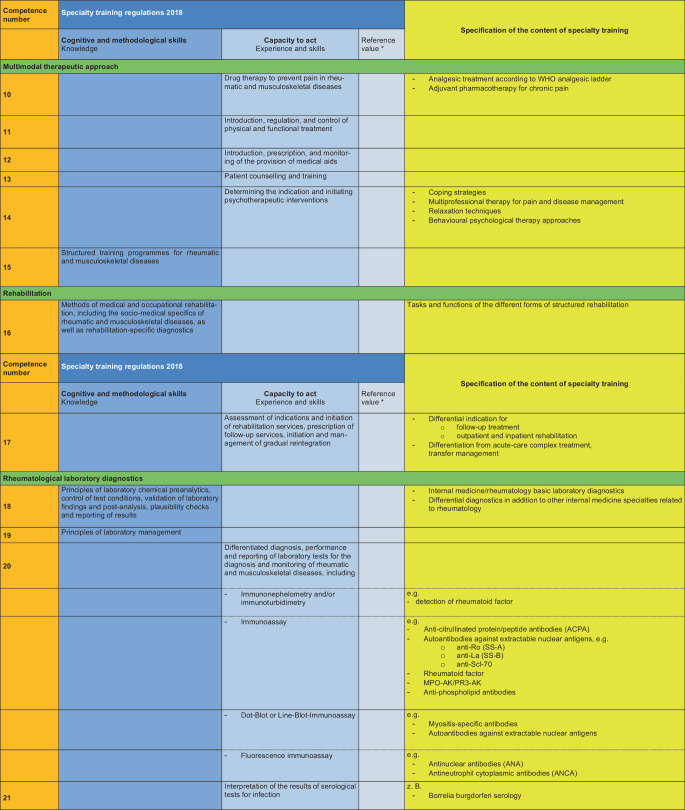
(Continued)

**Fig. 2:**
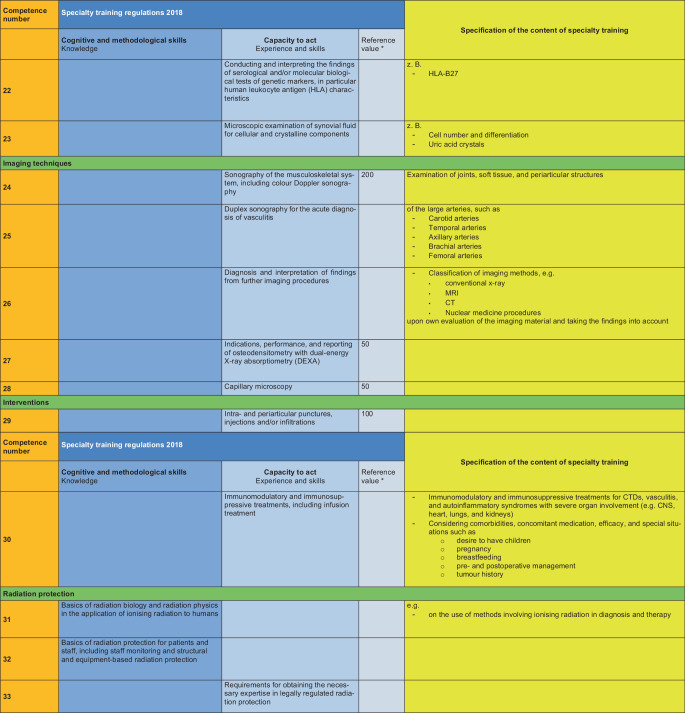
(Continued)

### III. Minimum criteria for the specific content of specialty training in internal medicine and rheumatology (see Table 1)

If all 33 competences (see Fig. 2 for details) can be fulfilled, authorisation should be granted for 36 months. If 25 out of 33 competences are fulfilled, whereby competence number 30 is considered mandatory, authorisation for specialty training can be granted for 24 months. An authorisation for specialty training for 12 months would be appropriate if at least 20 out of 33 competences are fulfilled.

**Table 1:** Minimum criteria for the specific content of specialty training in internal medicine and rheumatology

Extent	Content
36 months	**All 33 competences met** **Competencies 3, 8 and 30 are mandatory:** *Competence 3:* Treatment of CTDs and vasculitis with severe organ involvement (e.g. CNS, kidney, lung or heart involvement) requiring complex invasive diagnostics (e.g. transbronchial biopsy, kidney biopsy, myocardial biopsy and right heart catheterisation) for the evaluation and objectification of organ involvement and treatment management *Competence 8:* Treatment of severe infections (e.g. sepsis) with the need for cardiovascular monitoring, daily intravenous antibiotic therapy, or oxygen administration under immunosuppressive or immunomodulatory therapy *Competence 30:* Immunomodulatory and immunosuppressive treatments for CTDs, vasculitis and autoinflammatory syndromes with severe organ involvement (e.g. CNS, heart, lungs and kidneys)
24 months	25 of 33 competences met Competence 30 mandatory
12 months	20 out of 33 competences met
*The graduation of the specialty training scope was developed by the Commission for Continuing Education and Training of the German Society for Rheumatology and Clinical Immunology*	

### Determining the annual performance figures

When granting authorisation for specialty training, performance figures are of particular importance and are evaluated by the State Chambers of Physicians. The performance figures or treatment cases of a training supervisor are usually calculated using the following formula:

$$\begin{aligned} &\frac{\begin{array}{@{}c@{}} \text{Annual case}/\text{power number of the}\\ \text{authorised representative} \end{array}}{\text{Number of\rule{0pt}{10pt} trainees}}\\&=\text{Annual performance\rule{0pt}{10pt} rating per trainee}\end{aligned}$$

This position paper deliberately avoids providing detailed performance figures, which could be used, if necessary, to determine the number of training positions at a training centre.

### Assignment of responsibility for specialty training (see Fig. 3)

The scope of the specialty training authorisation to be granted is determined by the extent to which the requirements placed on the content, process and objectives of the specialty training can be fulfilled by the training supervisors, considering the care mandate, the performance statistics, and the personnel and material resources of the specialty training institution. This results in the competences to be taught at the training centre, which serve to grant the authorisation for specialty training in terms of scope and graduation of content.

**Fig. 3:**
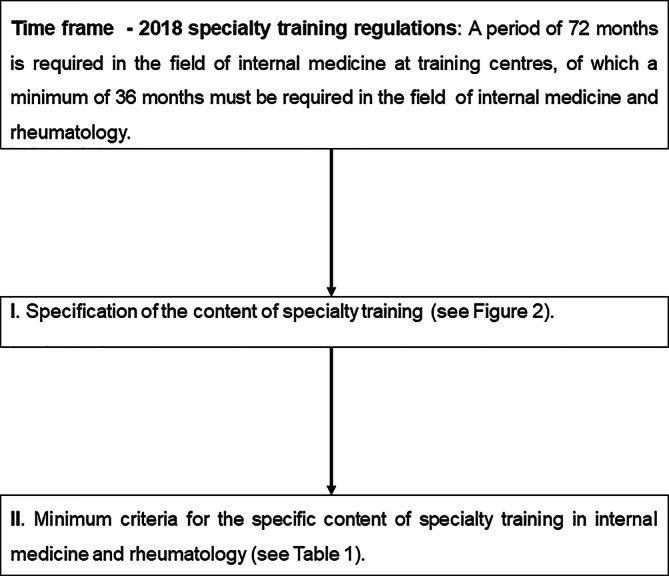
Flowchart of criteria for granting authorisation for specialty training in the field of internal medicine and rheumatology

## Discussion

Based on the 2018 model specialty training regulations in Germany, the State Chambers of Physicians developed chamber-specific specialty training regulations for those undergoing specialty training in the field of internal medicine and rheumatology to become specialists in internal medicine and rheumatology [4]. The contents of the MWBO for specialty training are specified in the FEWP, which provides guidelines for acquiring competencies for those undergoing specialty training and those authorised to provide specialty training [4, 5]. The model curriculum published in 2021 by the German Society for Rheumatology and Clinical Immunology (*Deutsche Gesellschaft für Rheumatologie und Klinische Immunologie,* DGRh) for specialty training in the field of internal medicine and rheumatology created a guideline for standardised teaching of core competencies as part of specialty training in internal medicine and rheumatology [10]. In contrast, there are currently no standardised criteria for training supervisors regarding the granting of training authorisation. With the publication of this position paper on the authorisation of specialty training, the DGRh is for the first time defining criteria for granting authorisation for providing specialty training in internal medicine and rheumatology. The presented catalogue of criteria opens up the possibility of a transparent and graduated granting of authorisation to provide specialty training in the field of internal medicine and rheumatology based on the skills taught and helps to ensure the quality of specialty training. As a basis for establishing criteria for the granting of authorisation for specialty training, 33 competences were defined, with a specification of the specialty training content based on the MWBO and the FEWP. Mandatory competences were defined and, based on the competences taught at the specialty training institution, a graduation of specialty training authorisations over 12–36 months was developed.

To date, specialty training to become a rheumatologist in Germany has mainly taken place in the inpatient sector [9], with inpatient training carried out in various types of hospitals (university hospitals, non-university hospitals and rehabilitation centres) and consequently involving different patient collectives of rheumatic disease. The possibility of specialty training also in private practice is also considered by the presented competence-based catalogue of criteria in a sectoral embracing structure of specialty training in rheumatology. This allows for assessment of the potential teaching skills for each training centre in a transparent way, independent of the care structure. The presented catalogue of criteria also allows for quick conclusions to be drawn regarding the missing skills of the specialty training institution, which can be acquired by the trainee at another specialty training institution through appropriate cooperation. In this regard, the present criteria catalogue also facilitates the establishment of collaborations between various training officers across the different sectors of patient care and enables the establishment of structured joint training programs. This is quite urgently needed in view of the existing shortage of specialists and the upcoming generational change [2, 6, 8], to attain optimal use of the existing out- and inpatient training capacities [11]. This approach also considers the cross-sectoral care of patients regarding specialty training, which is being pursued at the federal level in Germany.

To grade the specialty training authorisation, the corresponding competences and spectra of inflammatory rheumatic diseases were defined in the criteria, considering organ involvement and complications, to ensure quality of training across the entire range of inflammatory rheumatic diseases at the training centres. This also considers the interests of trainees, who demand the knowledge transfer through structured training and specialty education programs [7, 12].

The DGRh strives for continuous further development of the criteria catalogue for the authorisation to provide specialty training for the specialist’s qualification in internal medicine and rheumatology. The Commission for Continuing Education and Training always welcomes comments or suggestions for improvement. In addition, in the future, the criteria for granting specialty training authorisation must be continuously adapted to reflect diagnostic and therapeutic developments in the field of internal medicine and rheumatology.

In view of the impending generational change in rheumatology and the shortage of specialists, it is particularly important [2, 3, 6] to attract motivated young physicians to the field by providing attractive specialty training. The DGRh model curriculum for specialty training in rheumatology and the present list of criteria for granting authorisation for specialty training provide guidelines that enable high-quality and attractive specialty training in internal medicine and rheumatology.

## Abbreviations

DGRh: Deutsche Gesellschaft für Rheumatologie und Klinische Immunologie; German Society for Rheumatology and Clinical Immunology
FEWP: Fachlich empfohlener Weiterbildungsplan; recommended specialty training plan
MWBO: Musterweiterbildungsverordnung; specialty training regulations

## Declarations

For this article no studies with human participants or animals were performed by any of the authors. All studies mentioned were in accordance with the ethical standards indicated in each case.
